# The Uses of Tar-Water in Obstetrical and Gynæcological Practice

**Published:** 1880-10

**Authors:** Joseph Eve Allen

**Affiliations:** Augusta, Ga.


ATLANTA
### Medical and Surgical Journal.

VOL. XVIII.] OCTOBER—1880. [No. 7.

### Original Communications.

### THE USES OF TAR-WATER IN OBSTETRICAL AND GYNÆCOLOGICAL PRACTICE.

BY JOSEPH EVE ALLEN, M.D., AUGUSTA, GA.

Read before the Doctors' Society of Augusta, Ga.

Tar-water has long been known in medicine, for more than a hundred years ago Dr. Berkeley, Bishop of Cloyne, published a treatise* upon its therapeutic uses. This is the first written record which exists on the subject, but it is certain that tar-water had been employed before this time, because the author states that he first learned of its medicinal virtues during his travels in America, where it was used as a popular remedy for man and beast.

Lewis, Cullens, Erasmus, Darwin, Lisfranc and other distinguished men since Dr. Berkeley's time, have advised the use of tar-water in the treatment of various diseases. To Dr. L. A. Dugas, of Georgia, is due its general introduction into Southern surgical practice; his extensive experience with tar-water, especially during the late war, having conclusively demonstrated its great value as an antipyogenic.

Tar-water is made, according to the United States Dispensatory, by adding one pint of wood tar to four pints of cold water, mixing thoroughly and shaking frequently

*Siris, A Chain of Philosophical Reflections and Inquiries Concerning the Virtues of Tar-Water, etc.; second edition, 1744.

during twenty-four hours, and then filtering the infusion. The German Pharmacopæia directs that it be prepared by mixing one part of tar with ten parts of boiling distilled water, and macerating with constant stirring for two days. The French Codex leaves the tar in contact with thirty parts of water for one day, then rejects this strongly acid solution, and macerates the tea again with thirty parts of water for eight or ten days.

Lefort has determined the amount of tar taken up by hot water to be two grammes to the liter*.

The solvent action of cold water is greatly interfered with by the large quantity of oily principle present in tar. Magnes-Lohens has shown, however, that a saturated solution (six grammes of extract to the liter of water) may be obtained by first dividing the tar by admixture with twice its weight of some inert material, such as saw-dust; the use of magnesia for this purpose is inadmissible on account of the formation of soluble salts.

When prepared according to these different methods—that of the United States Dispensatory being, perhaps, the best—tar-water is a light brown liquid of a sharp, pungent taste, smoky odor and acid re-action; it is principally water—holding in solution a small proportion of pyroligneous acid, empyreumatic oil, creasote and resinous matters. If coal tar be substituted for wood tar in the preparation of tar-water the product is much the same, except that it contains carbolic acid instead of creasote.

Internally administered, tar-water is stimulant and diuretic in its action, and locally applied it is slightly astringent, unirritating and alterative; it is antiseptic and disinfectant, and hence antipyogenic, for by destroying the putrefactive germs it prevents or restrains the process of suppuration.

In calling attention to the uses of tar-water in obstetrical and gynæcological practice it is not expected to present anything especially new or original, but simply to report the good results which have attended its employment, in the hope that its value may become more generally recognized by the profession.

---

*National Dispensatory, page 754.

*Tar-water as an antiseptic during the puerperal state.*—Puerperal women, says Prof. Lusk*, tremble in the balance between a physiological and a pathological condition. The greatest danger to which they are exposed is septicæmia, to the development of which the state after delivery is peculiarly favorable, for the separation of the placenta leaves an extensive surface of the uterus unprotected by its lining membrane, and the bruised and lacerated tissues of the parturient passages are for days bathed in the lochial discharge—a fetid fluid consisting of the dissolved remains of blood-clots, pieces of placenta, shreds of membrane and other putrescent material. The absorption of this or any other decomposing matter is followed by some one of the manifestations of septicæmia. Autoinfection does not often occur because the lochial discharge does not begin to putrefy until after the placental site has become obliterated by the uterine contractions and the lacerations about the cervix and vulva have either healed by first intention or begun to granulate, for Billroth has shown that after healthy granulation is fairly established absorption of putrid matter goes on so slowly, if at all, that the poisoning of the blood does not occur, and that hence, for infection to take place, a fresh wound is required through which the septic material can enter the system. In exceptional cases, however, autoinfection may happen at the time of delivery, or immediately afterwards, before the wounds granulate, as when a putrid fœtus is expelled, or when the maternal soft parts have been so long subjected to pressure that sloughing is induced, or when from some diseased state of the genital organs, such as carcinoma, decomposing matter is already present at the time labor begins.

The clinical observations of Dr. Fordyce Barker† certainly prove that blood-poisoning may also result without traumatism being necessarily antecedent from septic material being formed within the system by certain morbid processes such as go on during the course of typhoid, scarlet and other fevers, and which give rise to disorganition and death of tissue, necrobiosis as it is called by Virchow.

*Transactions of the International Medical Congress, 1876, page 833.
†Puerperal Diseases, page 402.

Septicæmia is often due to decomposing matter being brought in contact with the recent wounds of the genitals by means of cloths, sponges, instruments and the hands of the accouchuer or attendants, and it is possible that septic material may even be conveyed to the puerperal woman by the air, but this method of infection is hardly probable.

The experiments of Pasteur and Tyndall have demonstrated that neither animal or vegetable substances can undergo putrifaction without the presence and rapid multiplication of living germs. When suppuration takes place these germs are met with in great numbers in the purulent discharges; they are variously termed microspores, micrococci or bacteria, and are always found in the air and water, and present different physical peculiarities which, in the higher forms, enable the microscopist to distinguish and classify them.

Chanvean and Burdon-Sanderson have proved that if a septic fluid be deprived of these bacteria by filtration it is no longer capable of producing toxæmia, though it may excite local inflammation; whereas the matter retained on the filter containing these organic germs possesses all the virulent properties of the original fluid; it does not follow from this, however, that bacteria are the sole sources of septicæmia, for it is possible that the septic poison and these germs may both be left upon the fitter. Pathologists regard bacteria as the cause of the morbid changes in septicæmia for the reason that, although all efforts to produce blood poisoning by isolated germs have as yet been attended with negative results, still the destruction of these germs in putrid material renders it innocuous, and they are constantly found in the infected wounds and throughout the diseased tissues. It is not known how bacteria induce disease in the human organism. Whether they produce a specific poison in the course of their growth and multiplication, or whether they simply act as carriers of the septic virus, or as mechanical irritants are questions to be settled by future pathological research.

Bacteria are always found on post-mortem examination in the tissues and fluids of the subjects of surgical pyæmia, erysipelas, diphtheria and puerperal septicæmia, and never found in those who have died of other diseases. This proves the existence of a common morbific element which binds these diseases due to septic infection together and establishes the connection which clinical observation has long shown to exist between them.*

The statement of Schroeder † cannot be accepted that puerperal fever is nothing else but the poisoning of the system with septic matter from the genital organs, because fever during the puerperal state may be dependent on a number of causes other than blood-poisoning, such as simple traumatic inflammation, zymotic and malarial influences and emotional disturbance, yet it is certain that that variety of puerperal fever, due to septicæmia, is the one most usually met with. Tyler Smith states that three thousand mothers die annually in England and Wales of puerperal fever and, according to Prof. Lusk, the mortality in New York city from this disease is one in every one hundred and forty-six confinements. When we consider therefore the great danger of septic infection to which the puerperal woman is exposed and the suffering and fatality which attends septicæmia in all of its different forms, we cannot too highly estimate the importance of using every means and agency to prevent its occurrence. The brilliant success of Listerism in surgery has, in England and Germany, led to its modified application in obstetrics with most satisfactory results. Thus for example, the method which Prof. Bischoff enforces in the Maternity of Basel,‡ and which has reduced the number of cases of septicæmia in that institution to an average of 16 per cent. for six years, requires the utmost cleanliness in the parturient woman and her surroundings. The use of antiseptic vaginal washes at the commencement and at intervals during the progress of labor, and the thorough disinfection of the hands before, and inunctions with car-

*Lusk on Puerperal Fever, Transactions International Medical Congress, 1876, page 841.

†Manual of Midwifery, page 331.

‡Address on Obstetrics by E. S. Lews, M.D., Transactions American Medical Association, 1879, page 229.

bolized oil during examinations. Directly after delivery the vagina is injected with a two or three per cent. solution of carbolic acid, and also the uterus, should its cavity have been entered by the hand as in cases of the removal of the placenta, turning or post partum hemorrhage. During the puerparel state vaginal injections of two per cent. solution of carbolic acid are used twice daily, or repeated every two hours in cases of difficult labor, putrid fœtus or retention of the ovum or placenta. Lacerations of the perineum are closed immediately with carbolized silk or cat gut sutures, and the vulva protected by a pad of picked lint soaked in a ten per cent. carbolized oil, which pad is frequently renewed.

The antiseptic management of labor and the puerperal state, as a matter of prophylaxis, is very important but unfortunately it is imposssible in private practice to carry out all its minute details, yet as far as possible they should be complied with.

The advantages which tar-water has over carbolic acid, chloride soda, thymol and other antiseptics, and which fit it especially for use during confinement, are: 1st. It is a perfect antiseptic and disisfectant, while its odor is pleasant and agreeable, and such as not to offend the most fastiaious. 2d. The oily and resinous principles which it contains exerts a healing action upon the genital lesions, and suppuration is prevented. 3d. The ease with which tar-water can be obtained and its great cheapness places it within the reach of the poorest people. Tar-water is to be used as a vaginal wash three times daily during the lying-in period, and the cloths used to protect the vulva and receive the discharges should be moistened with it. It may also be employed, should occasion demand, as a wash for the uterine cavity.

*The value of tar water as a local application in the treatment of certain diseases of the vulva, vagina and bladder, and as an antiseptic and disinfectant after operations in gynæcological surgery.*—Pruritus vulvæ is one of the most distressing affections peculiar to women, being usually not in itself a disease, but symptomatic of a number of diseases of the female genito-urinary apparatus; it is frequently met with in practice, and is often very obstinate and rebellious to treatment. Dependent upon so many and different causes no single remedy can be expected to prove efficacious in every case. The first effort of the physician should always be, therefore, to find out in each instance the pathological condition which produces the itching, and try to remove it by proper therapeutic measures. Often, however, the etiology of the affection is so obscure and the distress of the patient so great that palliative treatment is immediately demanded.

When, as is sometimes the case, itching of the vulva is a pure neurosis, a hyperaesthesia or neuralgia of the pudendal nerves, it is, of course, to be treated by remedies directed especially to the nervous system. This form of the affection is most usually met with during the early months of pregnancy, when, owing to the hyperæmia of these parts, all the pelvic organs are in a state of nervous etherism. Sub acute inflammation of the vulva induced either by irritating discharges or eruptive disorders is most frequently the cause of pruritu—thus it is according to Dr. Parnes, one of the earliest symptoms of uterine cancer, and it often occurs in the latter months of pregnancy caused by the acrid and profuse leucorrha which is present at the time. Itching is also met with in cases of chronic cervical and corporeal endometritis and vaginitis, the most obstinate form being that occurring after the menopause, caused by the scanty discharge of senile endometritis. During the course of diabetes, pruritus vulvæ is a common symptom due, as Dr. Thomas has shown, to the local action of the altered urinary secretion. Certain skin diseases such as eczema, prurigo, lichen and scabies—when occurring on the vulva, give rise to itching just as when seated elsewhere.

As excessive and acrid secretion is in the great majority of cases of pruritus vulvæ the exciting cause of the nervous irritation, we have in tar war a remedial agent which, used in connection with the other treatment appropriate to the case in hand, subserves a most valuable purpose. Its use in such cases as a vaginal injection and lotion to the vulva, neutralizes and renders innocuous the irritating discharges, and by its sedative and alterative action it restrains or stops the morbid process. When the pruritus is due to skin diseases, the benefit derived from the use of tar-water is readily understood in view of the long recognized curative value of tar preparations in the treatment of these affections in other parts of the body.

In acute and chronic catarrhal vaginitis, tar-water used either alone or as a vehicle for other medicinal substances is of the greatest benefit; its disinfectant and astringent properties render it especially valuable in gonorrhœal vaginities. In the latter months of pregnancy women frequently suffer from a profuse and very irritating leucorrhœa, which comes from the upper part of the vagina, and is due to the congestion of the mucous membrane, which presents granulations similar to that observed in cases of vaginitis from contagion. Doubtless in very severe cases of this affection the cauterization of the cervix, as advised by Dr. Henry Bennett, is necessary to effect a cure, but generally washing out the vagina with warm tar-water twice daily, is attended with such amelioration in the condition of the patient that the end of gestation may be awaited without resort to more active treatment.

Leucorrhœa, great in quantity and purulent in character, is often met with in young girls, due to inflammation of the vulva, caused most frequently by uncleanliness, though sometimes by the presence of ascarides, and by reflex irritation from the teeth and digestive organs. Constitutional influences such as the strumous diathesis are of great etiological importance, the discharge in such cases being of the nature of the otorrhœa, which is also common to these subjects. That uncleanliness is, however, the most usual cause of this affection is certain. Mr. Bouchut has shown* that the pudendal gland in the female, like the preputial glands in the male, furnish a secretion which, when allowed to accummulate, invariably causes irritation, followed by catarrhal inflammation. In cases of this affection, frequent ablutions with tar-water, together with the internal administration of tonics and alteratives

Gazette Obstetrical, June 20, 1880.

are rapidly curative. After each washing a pledget of lint soaked in tar should be placed between the labia.

In the treatment of various uterine diseases hot tar-water vaginal irrigations, after the method of Dr. Emmet, is of great benefit.

Prof. Skene mentions* tar-water internally administered as a remedy for chronic cystitis. In this disease the daily injection of the bladder with hot tar water in connection with appropriate internal treatment, is often attended with the most marked and rapidly beneficial results. By its use the decomposition of the urine in the bladder is perfectly prevented, and the cure of the inflammation of the mucous membrane, as evinced by the subsidence of vesical irritability, tenesmus and the stopping of the secretion of purulent mucous, is soon effected.

After operations in gynæcological surgery, tar-water has been found to be equal to carbolic acid solution in value as an antiseptic and disinfectant, while its greater virtue as an antipyogenic makes it more efficacious in preventing suppuration.

